# C2GAP2 is a common regulator of Ras signaling for chemotaxis, phagocytosis, and macropinocytosis

**DOI:** 10.3389/fimmu.2022.1075386

**Published:** 2022-11-29

**Authors:** Xuehua Xu, Henderikus Pots, Bernd K. Gilsbach, Dustin Parsons, Douwe M. Veltman, Sharmila G. Ramachandra, Haoran Li, Arjan Kortholt, Tian Jin

**Affiliations:** ^1^ Chemotaxis Signaling Section, Laboratory of Immunogenetics, National Institute of Allergy and Infectious Diseases, National Institutes of Health, Rockville, MD, United States; ^2^ Department of Cell Biochemistry, University of Groningen, Groningen, Netherlands; ^3^ Functional Neuroproteomics and Translational Biomarkers in Neurodegenerative Diseases German Center for Neurodegenerative Diseases (DZNE), Tübingen, Germany

**Keywords:** macropinocytosis, phagocytosis, G protein coupled receptor, chemotaxis, model organism *dictyostelium*, ras, GTPase activating proteins (GAPs)

## Abstract

Phagocytosis, macropinocytosis, and G protein coupled receptor-mediated chemotaxis are Ras-regulated and actin-driven processes. The common regulator for Ras activity in these three processes remains unknown. Here, we show that C2GAP2, a Ras GTPase activating protein, highly expressed in the vegetative growth state in model organism *Dictyostelium*. C2GAP2 localizes at the leading edge of chemotaxing cells, phagosomes during phagocytosis, and macropinosomes during micropinocytosis. *c2gapB−* cells lacking C2GAP2 displayed increased Ras activation upon folic acid stimulation and subsequent impaired chemotaxis in the folic acid gradient. In addition, *c2gaB^-^* cells have elevated phagocytosis and macropinocytosis, which subsequently results in faster cell growth. C2GAP2 binds multiple phospholipids on the plasma membrane and the membrane recruitment of C2GAP2 requires calcium. Taken together, we show a shared negative regulator of Ras signaling that mediates Ras signaling for chemotaxis, phagocytosis, and macropinocytosis.

## Introduction

The model organism *Dictyostelium discoideum* is a free-living professional phagocyte. It eats bacteria as a food source through phagocytosis. It also grows in axenic culture medium by engulfing liquid nutrients through macropinocytosis (1). *D. discoideum* grows and divides as separate, independent cells in the growth stage (vegetative stage). Environmental changes, such as starvation, initiate development of *D. discoideum* (social stage). Some cells start to secrete cAMP, the first identified chemoattractant in *D. discoideum*. Neighboring cells sense cAMP by the G protein coupled receptor (GPCR) cAR1 and move toward the source of cAMP through chemotaxis. cAMP-mediated chemotaxis in *D. discoideum* represents the best-studied system in eukaryotic cell chemotaxis. Thus, *D. discoideum* has been extensively used as a model organism to study GPCR-mediated chemotaxis, phagocytosis, and macropinocytosis, three fundamental processes play pivotal roles in innate immunology. Ras plays central roles in these three processes (2–4). However, the common regulator of Ras signaling in these three processes remain unknown.

Ras signaling is activated by guanine nucleotide exchange factors (GEFs) and deactivated by GTPase-activating proteins (GAPs). *D. discoideum* encodes 15 Ras subfamilies, 26 RasGEFs, and 17 RasGAPs. Cells deficient in RasB, RasG, RasS, or Rap1 mutation display decreased macropinocytosis and phagocytosis, suggesting that these proteins play essential roles in these two processes (2, 5–9). It has also been shown that GefB and GflB play a critical role in macropinocytosis and phagocytosis (8, 10, 11). Several RasGAPs have been found to deactivate Ras signaling in these two processes. NF1, IQGC, and RGBARG play roles in deactivating Ras activity in macropinocytosis and phagocytosis (12–14). DdNF1 and C2GAP1 are essential for cAMP-mediated Ras adaptation and chemotaxis during the early developmental stage (15–17). Importantly, *D. discoideum* also senses folic acid, a second chemoattractant secreted by bacteria, and moves toward the source (bacteria) through chemotaxis and eventually phagocytoses the bacteria when in the vegetative stage (18). Recently, the receptor of folic acid, FAR1, has been identified (4). Folic acid stimulates the G protein coupled receptor FAR1 to activate heterotrimeric Gα4Gβγ to control signaling pathways of chemotaxis (4, 19, 20). Folic acid stimulation also triggers a transient Ras activation (21). Folic acid-induced Ras activation was significantly reduced in cells lacking RasG or RasC/G, suggesting that RasC and RasG might be the major Ras isoforms to be activated by folic acid. The negative regulator of Ras signaling in folic acid-mediated chemotaxis remains unknown. More importantly, the common negative regulator of Ras signaling in these three fundamental processes remain elusive. In the present study, we identified C2GAP2, a highly expressed RasGAP protein in vegetative stage, that regulates folic acid-mediated chemotaxis, phagocytosis, and macropinocytosis in *D. discoideum*. This thus suggests that C2GAP is a common negative regulator of Ras signaling in these three fundamental processes.

## Results

### C2GAP2 is a Ras GAP protein highly expressed in the vegetative stage of *D. discoideum*

**Figure 1:**
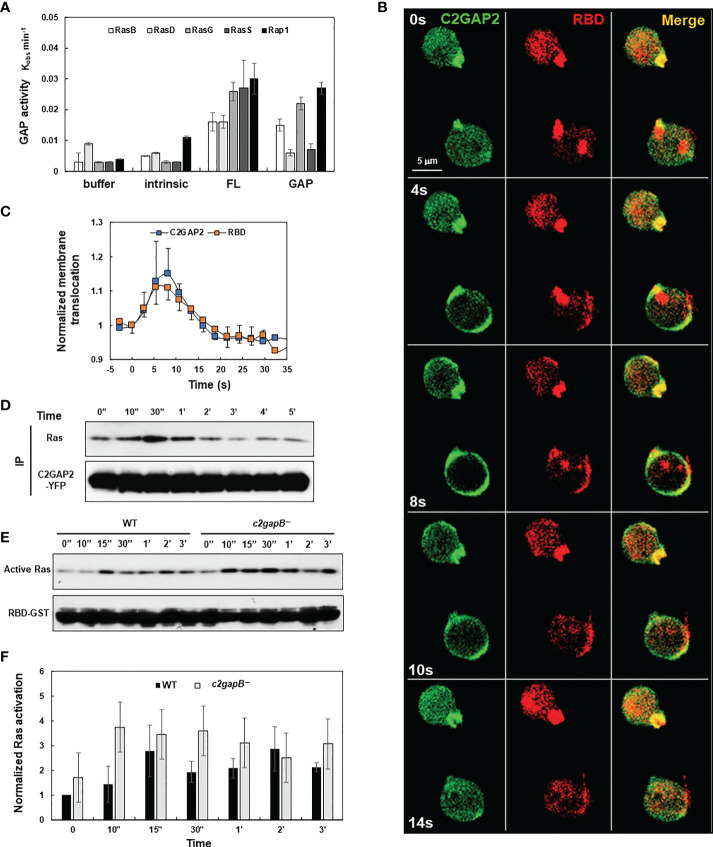
C2GAP2 controls folic acid-induced Ras adaptation. **(A)** GAP activity of C2GAP2 for Ras isoforms. **(B)** Montage shows folic acid-induced membrane translocation of C2GAP2 and RBD-RFP (active Ras binding domain-tagged with RFP). Cells expressing C2GAP2-YFP and active Ras probe (RBD-RFP) were stimulated with 100 μM folic acid at time 0 s. **(C)** Graph shows the dynamics of folic acid-induced membrane translocation dynamics of C2GAP2 and active Ras upon folic acid stimulation. **(D)** A co-immunoprecipitation analysis shows that folic acid stimulation induces the association between Ras and C2GAP2. Cells expressing C2GAP2-YFP were stimulated with 100 μM folic acid at time 0, and cells were collected and lysed at the indicated time points. Lysates were incubated with agarose beads coupled with anti-GFP antibody and elutes were analyzed by immunoblotting to detect Ras and C2GAP2-YFP using anti-pan Ras (top panel) and anti-GFP (bottom panel) antibodies, respectively. **(E)** Folic acid-induced Ras activation in wild-type (WT) and *c2gapB−* cells determined by a pull-down assay. Upon stimulation with 10 μM folic acid at time 0, cells were collected and lysed at the indicated time points. Lysates were incubated with agarose beads coupled with RBD-GST (active Ras binding domain tagged with GST) and elutes were analyzed by immunoblotting with anti-pan Ras antibody (top panel) and anti-GST antibody (bottom panel). **(F)** Normalized quantitative densitometry of the active Ras in **(E)** The intensity ratio of the active Ras in WT at time 0 s was normalized to 1. Mean ± SD from three independent experiments is shown.

C2GAP2 (gene ID: DDB0205121 and gene name *c2gapB*) is a Ras GAP protein, which contains one C2 and one GAP domain (15, 22). *c2gapB* was highly expressed in the vegetative stage and displayed a decreasing expression pattern during the early development of *D. discoideum* (**Figure S1**). It possessed GAP activity toward the main Ras isoforms that play major roles in diverse cellular processes in the vegetative stage of *D. discoideum* (**Figure 1A**). It localized in active Ras-enriched protrusion sites of resting cells (**Figure 1B**). Folic acid stimulation triggered robust translocation of C2GAP2 to the plasma membrane (PM), where it colocalized with an active Ras probe (RBD-RFP, active Ras-binding domain of Raf1 tagged with RFP). The PM-translocating dynamics of these two proteins were similar (**Figure 1C**). Consistent with the above, folic acid stimulation also promoted the association between C2GAP2 and Ras (**Figure 1D**). To understand the role of C2GAP2 in Ras activation, we generated stable cell lines deficient in C2GAP2 (*c2gapB−*) (**Figure S2**). We then measured the Ras activation profile in wild-type (WT) and *c2gapB−* cells in response to folic acid. Folic acid stimulation triggered Ras activation in the vegetative *D. discoideum* WT cells, while it induced an elevated Ras activation in *c2gapB−* (**Figures 1E, F**). The above results indicate that C2GAP2 functions as a Ras GAP protein that deactivates active Ras in the vegetative stage of *D. discoideum*.

### C2GAP2 is required for folic acid receptor (FAR)-mediated chemotaxis

**Figure 2:**
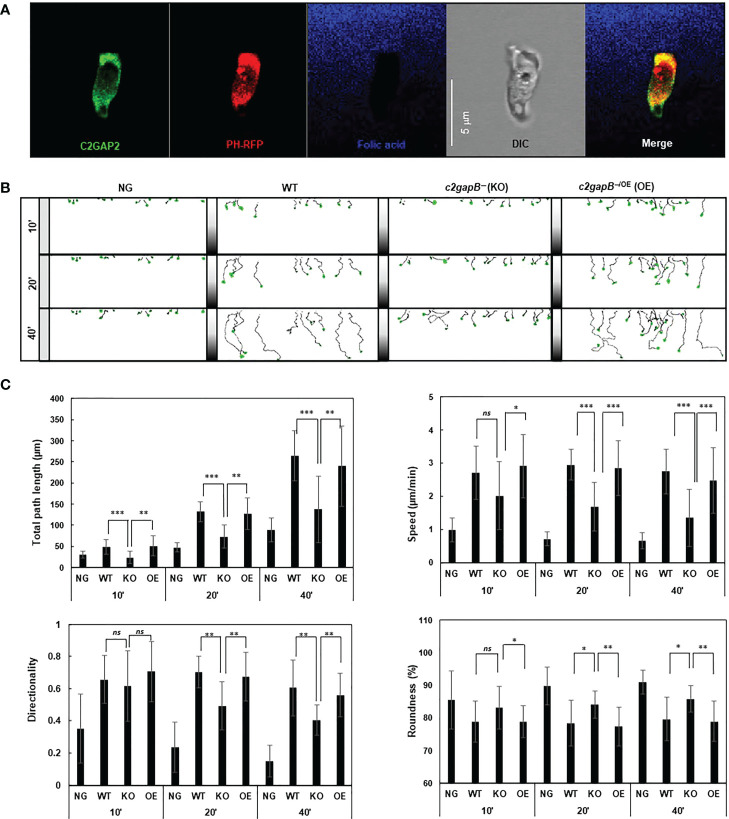
*c2gapB−* cells display impaired chemotaxis in a folic acid gradient. **(A)** Leading edge localization of C2GAP2 in chemotaxing cells. Cells expressing C2GAP2-YFP (green) and the PIP_3_ biosensor PH_Crac_-RFP (PIP3-binding domain of Crac tagged with RFP, red) were chemotaxing a folic-acid gradient (blue). To visualize the gradient, 125 μM folic acid was mixed with fluorescent dye Alexa 633 and released from a microinjector. **(B)** Montages show the traveling path of chemotaxing cells of WT, *c2gapB−* (KO), *c2gapB−* expressing C2GAP2-YFP (*c2gapB−* ^/OE^ or OE) in no gradient (NG) or a folic acid gradient (100 μM) for 10’, 20’, or 40’. Cell migration was monitored using EZ-TAXIScan. **(C)** Chemotaxis behaviors measured from B are described by four parameters: directionality, specifically “upward” directionality, where 0 represents random movement and 1 represents straight movement toward the gradient; speed, defined as the distance that the centroid of the cell moves as a function of time; total path length, the total distance the cell has traveled; and roundness (%) for polarization, which is calculated as the ratio of the width to the length of the cell. Thus, a circle (no polarization) is 1 and a line (perfect polarization) is 0. Twenty-five cells in each group were analyzed using DAIS software (24). Mean ± SD is shown. The *p* values of Student’s *t*-test are indicated as *ns* (not significant, *p* > 0.1), *(*p* < 0.1), **(*p* < 0.01), or ***(*p* < 0.001).

Adaptation is a fundamental strategy by which eukaryotic cells to chemotax through chemoattractant gradients with a large concentration range (15, 16, 23). The above data show that in response to folic stimulation, *c2gapB−* cells displayed failure in Ras adaptation (**Figures 1D, E**). In addition, we found that C2GAP2 localized in the leading edge of chemotaxing cells in a folic acid gradient (**Figure 2A**), indicating its potential role in gradient sensing and maintaining the polarization during chemotaxis. Hence, we examined the chemotaxis behaviors of WT, *c2gapB−* cells and *c2gapB−* cells epigenetically expressing C2GAP2-YFP (*c2gapB−/OE*) in the gradients of folic acid (**Figure 2B**). We found that, comparing to WT cells, *c2gapB−* displayed impaired chemotaxis while *c2gapB−/OE* showed a normal chemotaxis, indicating that C2GAP2 expression restores the chemotaxis capability in *c2gapB−* cells. *c2gapB−* cells that migrated in a folate acid gradient for a longer time and experienced gradient at a higher concentration showed a more severe defect in chemotaxis (**Figure 2C**). Taken together, C2GAP2 plays an important role in the folic-acid mediated chemotaxis.

### C2GAP2 localizes to phagosome and plays a negative role in phagocytosis

**Figure 3:**
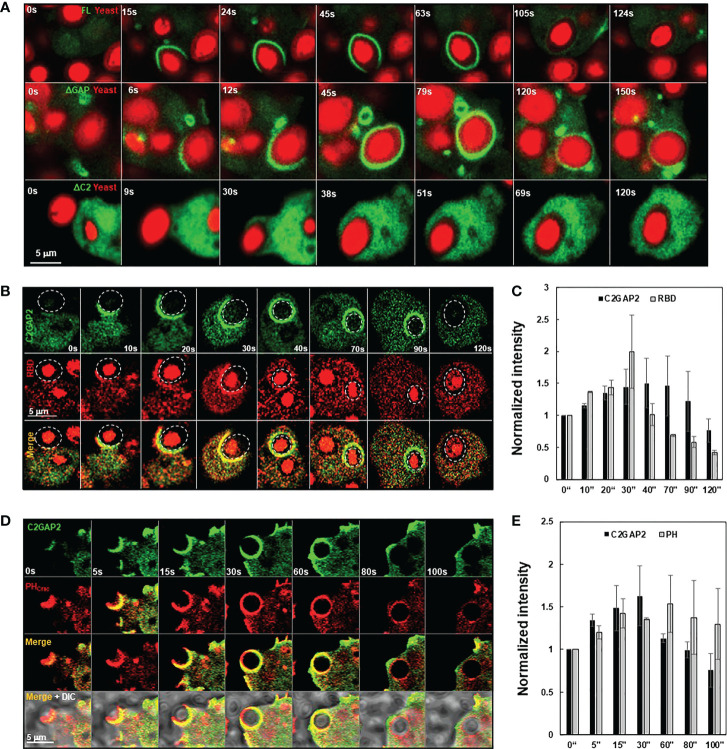
Cellular localization of C2GAP2 during phagocytosis. **(A)** Localization of C2GAP2 in a phagosome requires its C2 domain. Cells expressing YFP-tagged FL, ΔC2, or ΔGAP (green) were monitored. The yeasts were killed and labeled with Alexa 594 (red). Montages and graphs show temporospatial localization of C2GAP2 and active Ras **(B, C)** and PIP_3_ **(D, E)** during phagocytosis. Cells expressed C2GAP-YFP and active Ras probe (RBD-RFP) in **(B)** or PIP_3_ probe (PH_Crac_-RFP) in **D**, respectively. The intensity of C2GAP2-YFP/RBD-RFP **(C)** or C2GAP2-YFP/PH_Crac_-RFP **(E)** at time 0” was normalized to 1. Mean ± SD was shown in **(C)** and **(D)** N = 3 in **(C)** and **(D)**, respectively.

C2GAP2 possessed RasGAP activity toward Ras isoforms that play essential role in phagocytosis. Thus, we monitored the cellular localization of C2GAP2 during phagocytosis (**Figure 3A**). Cells expressing C2GAP2-YFP (green) were incubated with yeast fluorescently labeled with Alexa 594 (red). C2GAP2-YFP localized to the phagocytic cup and phagosome, and then gradually left the phagosome (**Figure 3A**, top panel). To understand the domain requirement for phagosome localization, we monitored the cellular localization of full- length C2GAP2 (FL) or deletion mutants, which lacks either the C2 domain (ΔC2) or the GAP domain (ΔGAP), during the phagocytosis of yeast (**Figure S3**). We found that the ΔGAP mutant maintained while the ΔC2 mutant lost localization on the phagocytic cup and phagosome (**Figure 3A**, middle and low panels), indicating that the C2 domain is required and sufficient for localization during phagocytosis. Next, we simultaneously monitored the temporospatial localization of C2GAP2-YFP and active Ras using RBD-RFP during phagocytosis (**Figure 3B**). We found that both active Ras and C2GAP2 colocalized on the initiation site of the phagocytic cup (0 s), on the phagocytic cup (10 – 30 s), and then on the phagosome (40 s). Interestingly, active Ras in the phagosome decreased and disappeared from the phagosomes (around 40 s), and C2GAP2 stayed and then gradually disappeared (40 to 90 s). Quantitative measurement of the temporospatial intensities of C2GAP2-YFP and RBD-RFP during phagocytosis confirms the above observation (**Figure 3C**). We further monitored the temporospatial localization of C2GAP2-YFP and phosphatidylinositol (3,4,5)-trisphosphate (PIP_3_) using a PIP_3_ biosensor, PH_Crac_-RFP (25). PIP_3_ is generated by PI3K, a direct effector of active Ras, and plays a critical role in phagocytosis (3, 26). We found that both C2GAP2 and PH-RFP colocalized on the initiation site of the phagocytic cup (0 s), on the phagocytic cup (10 – 30 s), and then on the phagosome (60 s) (**Figure 3D**). C2GAP2 in phagosome decreased and disappeared (around 40 s), and PH-RFP stayed and then gradually disappeared (60 to 100 s). C2GAP2 was enriched at closing sites during the closure of the phagocytic cup to the phagosome, indicating its role in this process. Quantitative measurement of C2GAP2-YFP and PH_Crac_-RFP during phagocytosis is shown in **Figure 3E**. Cellular localization of C2GAP2 at the initiation sites of phagocytic cup and phagosome indicates its potential role in regulating Ras activity during phagocytosis.

**Figure 4:**
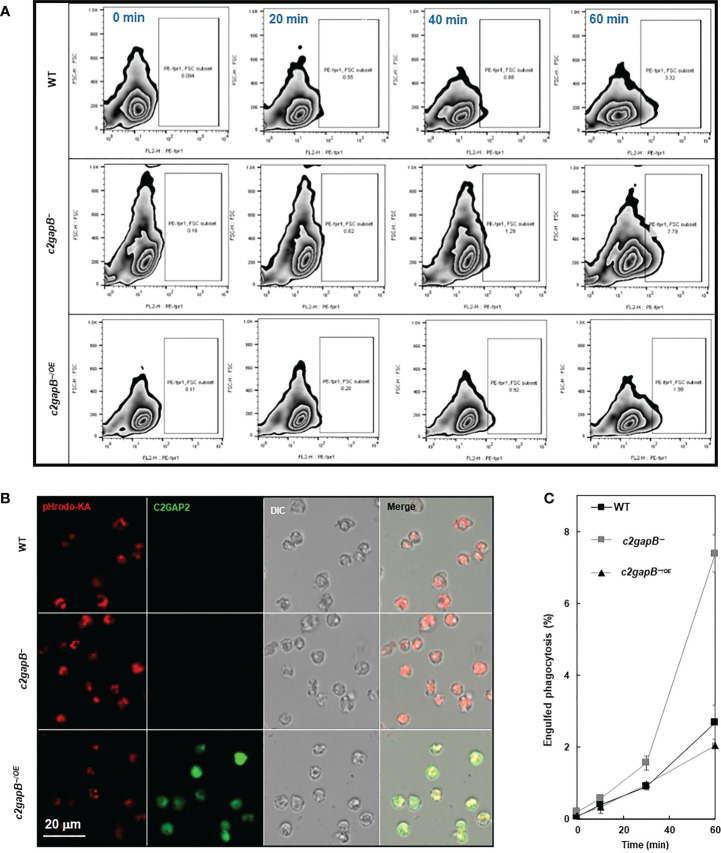
C2GAP2 plays a negative role in phagocytosis. **(A)** Bacterial phagocytosis of WT, *c2gapB−*, and *c2gapB−* ^/OE^ cells. Cells were mixed with pHrodo-labelled live *Klebsiella aerogenes* at a 1:50 ratio for the indicated time. Cells were suspended in basic pH buffer and analyzed for the percentage of pHrodo-positive cells, which represents the cells that engulfed *K. aerogenes*. **(B)** Graph shows mean ± SD from three independent repetitions of the experiments exemplified in **(A)**. **(C)** Montage shows the cells of WT, *c2gapB−*, and *c2gapB−^/OE^* cells, which were mixed with pHrodo-labeled *K. aerogenes* at a 1:50 ratio for one hour. Next, cells were mounted on a slide in basic pH buffer and analyzed by confocal microscopy. The engulfed pHrodo-labelled *K. aerogenes* are shown as red.

To understand the role of C2GAP2 in phagocytosis, we compared bacterial phagocytosis in WT, *c2gapB−*, and *c2gapB−* ^/OE^ cells (**Figure 4**). Cells were mixed with pHrodo-labelled live *Klebsiella aerogenes* at a rate of 1:50 and sampled at the indicated time points. The phagocytosed *K. aerogenes* was measured by flow cytometry as red fluorescent signal in the cells (**Figure 4A**). We also visualized the phagocytosed bacteria in the cells at 60’ using confocal microscopy and detected a notable higher bacterial phagocytosis (red) in *c2gapB−* cells and a reduced phagocytosis in *c2gapB−* ^/OE^ cells, in comparison to WT cells (**Figure 4B**). Quantitative measurement of three independent experiments confirms the above observation (**Figure 4C**). The above result indicates a negative role of C2GAP2 in phagocytosis.

### C2GAP2 localizes to the macropinosome and plays a negative role in macropinocytosis and subsequent axenic cell growth

**Figure 5:**
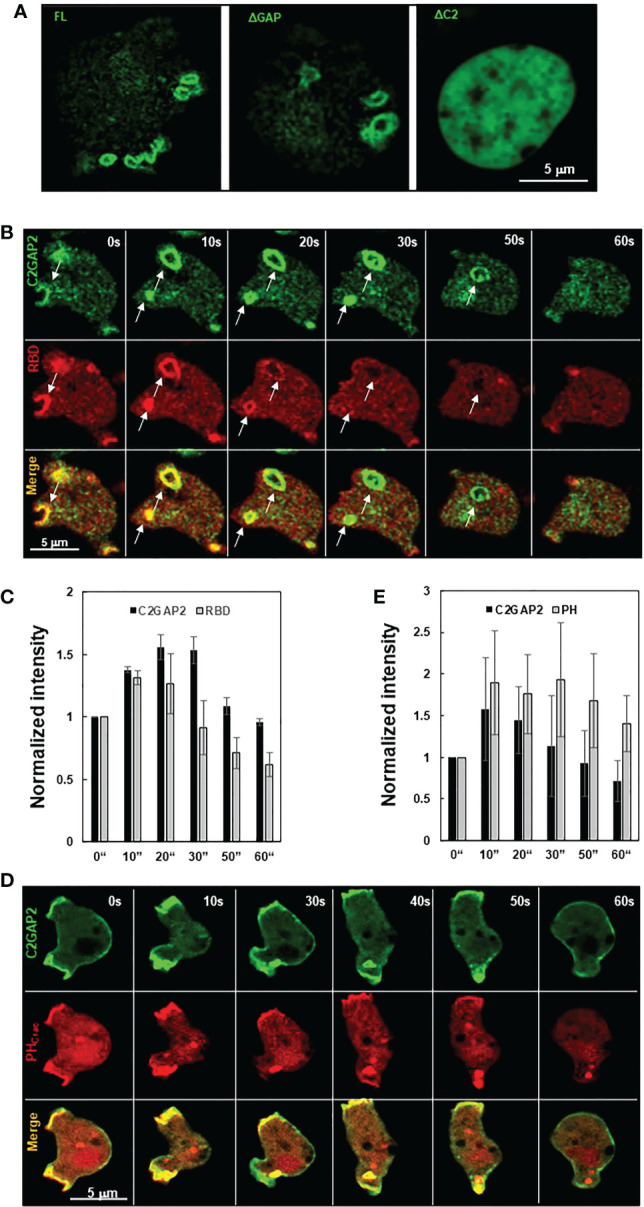
Cellular localization of C2GAP2 during macropinocytosis. **(A)** Localization of C2GAP2 in macropinocytosis requires its C2 domain. Cells expressing YFP tagged full-length or deletion mutants of C2GAP2 (green) were monitored. Montage **(B)** and graph **(C)** show temporospatial localization and quantitative measurement of C2GAP2 and active Ras during macropinocytosis. Cells expressing both C2GAP-YFP (green) and active Ras probe (active Ras binding domain of human Raf1 tagged with RFP, RBD-RFP, red) were monitored. Montage **(D)** and graph **(E)** show temporospatial localization of C2GAP2 and PIP_3_ during macropinocytosis. Cells expressing C2GAP-YFP (green) and the PIP_3_ probe, PH_Crac_-RFP (red), were monitored. The intensity of C2GAP2-YFP/RBD-RFP **(C)** or C2GAP2-YFP/PH_Crac_-RFP **(E)** at time 0” was normalized to 1. Mean ± SD was shown in **(C)** and **(D)** N = 3 in **(C)** and **(D)**, respectively.

*D. discoideum* cells engulf fluidic nutrients through macropinocytosis, a cellular process regulated by Ras activation (1, 2). Ras activation at membrane patches is essential to induce macropinosomes and the loss of the RasGAPs at these membrane ruffles potentiates Ras activation and subsequent macropinocytosis, while their overexpression repress macropinocytosis (1, 5, 13). We found that C2GAP2 localized in the macropinosome in the cells with culture medium (**Figure 5A**). The ΔGAP mutant maintained while the ΔC2 mutant lost localization on the macropinosome, indicating that the C2 domain is required and sufficient for the localization. Next, we monitored the temporospatial localization of C2GAP2 (green) and RBD-RFP (red) during macropinocytosis (**Figure 5B**). We found that C2GAP2 colocalized with active Ras on the membrane ruffles (0 s), which often further close to form macropinosomes (10 s). The amount of active Ras decreased (20 s), while C2GAP2 maintained its localization on the macropinosome (30 s), then gradually decreased (50 s) and completely disappeared around 60 s. Quantitative measurement of temporospatial intensities of C2GAP2-YFP and RBD-RFP during macropinocytosis is shown in **Figure 5C**. We further monitored the temporospatial localization of C2GAP2-YFP and PIP_3_ using PH_Crac_-RFP (**Figure 5D**). Both C2GAP2 and PH_Crac_-RFP colocalized on the initiation site of the macropinocytic cup (0 s) and the macropinosome (10-40 s). Then, the localization of C2GAP2 in phagosome decreased and disappeared (40 to 60 s), while PH_Crac_-RFP still localized and then gradually disappeared (after 60 s). Quantitative measurement of temporospatial intensities of C2GAP2-YFP and RBD-RFP during macropinocytosis is shown in **Figure 5E**. The localization of C2GAP2 at the initiation sites of the macropinocytic cup and the macropinosome indicates its potential role in regulating Ras activity during macropinocytosis.

**Figure 6:**
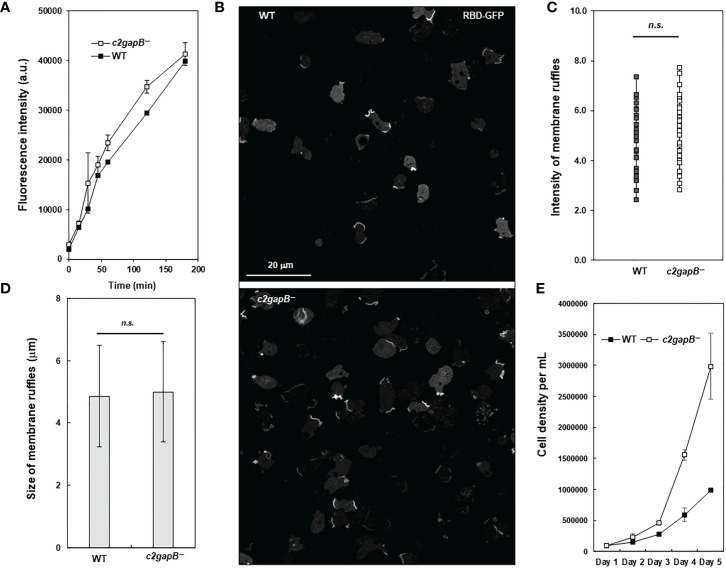
C2GAP2 is involved in macropinocytosis. **(A)** Fluid phase endocytosis of vegetative cells in shaken suspension was determined using uptake of fluorescent FITC-dextran. **(B)** Montage shows active Ras labeled by RBD-GFP at the membrane ruffles that often close to form macropinosomes in WT and *c2gapB−* cells. **(C, D)** RBD-GFP patches from cells in panel **(B)** were quantified using image analysis. The intensity and size of RBD-GFP patches at membrane ruffles were quantified as a readout of Ras activation levels in macropinosomes. The *p* values of Student’s *t*-test are indicated as not significant (*ns* if *p* > 0.1). **(E)** Axenic cell growth of WT and *c2gapB−* cells in the suspension culture. Mean ± SD of cell density from three independent cell cultures is shown.

To understand the role of C2GAP2 in macropinocytosis, we compared the uptake of fluorescent FITC-dextran in vegetative WT and *c2gapB−* cells in shaken suspension and found an increased macropinocytosis in *c2gapB−* cells as previously described (**Figure 6A**). We further quantitatively measured the intensity and size of membrane ruffles in both WT and *c2gapB−* cells as previously described (27). We found no significant differences in the size or the intensity of macropinosomes in WT and *c2gapB−* cells (**Figures 6B, D**), indicating that C2GAP2 plays no essential role in controlling the size and the maturation of macropinosome, instead, plays a role in the speed of macropinocytosis. To determine the consequence of the increased macropinocytosis, we next measured axenic cell growth of WT and *c2gapB^-^* cells in suspension culture (**Figure 6E**). We found an increased cell growth in *c2gapB−* cells. Taken together, the above results indicate a negative role of C2GAP2 in macropinocytosis and consequent cell growth.

### Molecular mechanism of C2GAP2 membrane targeting

**Figure 7:**
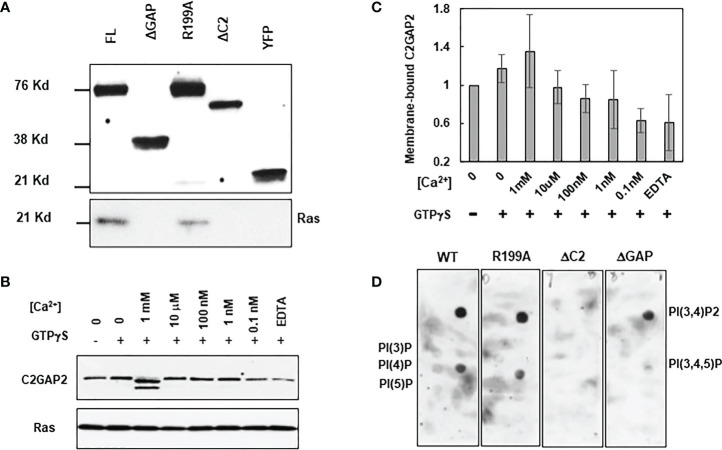
Molecular mechanism of targeting C2GAP2 to the plasma membrane. **(A)** A co-immunoprecipitation analysis indicates the requirement for Ras and C2GAP2 interaction. Cells expressing full-length (FL) or deletion mutants of ΔGAP or ΔC2 of C2GAP2 tagged with YFP were stimulated with 100 μM folic acid for 30 s and lysed. Lysates were incubated with agarose beads coupled with anti-GFP antibody and elutes were analyzed by immunoblotting to detect Ras and C2GAP2-YFP using anti-pan Ras (top panel) and anti-GFP (bottom panel) antibodies, respectively. **(B)** Calcium promotes the membrane localization of C2GAP2. Cells expressing C2GAP2-YFP were lysed with present or without GTPγS or [Ca^2+^] at the indicated concentrations. Membrane fractions were collected and subjected to Western blot detection. Anti-GFP (top panel) antibodies detects membrane-bound C2GAP2-YFP, and anti-pan Ras (lower panel) detect Ras as a control of the membrane protein and the C2GAP2-interacting protein. **(C)** Quantification of calcium-dependent membrane targeting of C2GAP2 shown in B and two other independent experiments. The intensity ratio of Ras and C2GAP2 without GTPγS and [Ca^2+^] was normalized to 1. **(D)** C2GAP2 binds mainly to two species of phospholipids on the membrane.

Proteins and phospholipids on the plasma membrane play critical roles in membrane targeting of C2 domain-containing RasGAPs (15, 28). Thus, we investigated the requirement of the C2 domain for C2GAP2’s interaction with Ras by immunoprecipitation analysis (**Figure 7A**). Cells expressing either YFP-tagged FL, ΔGAP, ΔC2, or inactive mutant R199A were stimulated with 100 μM folic acid for 30 s and lysed. YFP-tagged proteins in the cell lysates were subjected to immunoprecipitation using anti-GFP (also anti-YFP) antibodies, which were pre-conjugated with agarose beads. Ras was detected from the cells expressing either FL or R919A mutant, but not from ΔGAP or ΔC2, indicating that the interaction between C2GAP2 and Ras requires both the GAP and C2 domains, but not GAP activity. The C2 domain often requires calcium for membrane targeting (28–30). Thus, the membrane fraction of cells expressing C2GAP2-YFP in the present or absence of GTPγS or [Ca^2+^] at the indicated concentrations was obtained through 5-μm filter units (**Figure 7B**). Ras was detected as the control for membrane protein. We found increased membrane localization of C2GAP2 in the presence of calcium, indicating that calcium binding plays a role in its membrane targeting (**Figure 7C**). It has been previously reported that several C2 domains bind to multiple phospholipids on the plasma membrane and this binding plays a role in the membrane targeting of C2 domain-containing protein (28). We therefore determined phospholipids on the plasma membrane using PIP Strips as previously reported (**Figure 7D**) (17). We found that C2GAP2 displayed strong binding with two phospholipids (PI(3,4)P_2_ and PI(3,4,5)P_3_) and relatively low binding with PI(3)P, PI(4)P and PI(5)P on the plasma membrane. Thus, calcium binding and the presence of Ras and appropriate phospholipids on the plasma membrane play a role in the membrane targeting of C2GAP2.

## Discussion

GPCR-mediated chemotaxis, phagocytosis, and macropinocytosis are mediated by Ras. In the current study, we demonstrated that C2GAP2 is a common regulator of Ras signaling in chemotaxis, macropinocytosis, and phagocytosis.

Multiple RasGAPs, including NF1, IqgC, and RGBARG, have been shown to be involved in regulating Ras signaling in macropinocytosis and phagocytosis (1, 13). NF1 localizes at the active Ras-enriched protruding sites and further extends and closes to form macropinosome and phagosome (1). Cells lacking NF1 (*axeB−*) generate larger-than-normal phagosomes and macropinosomes, which enable *D. discoideum* to grow in axenic culture medium. RGBARG is a multidomain protein containing a RCC1, a RhoGEF, a BAR, and a RasGAP domain (13). RGBARG uses a tripartite mechanism of Ras, Rac, and phospholipid interactions to localize at the protruding edge and interface with the interior of both macropinocytic and phagocytic cups. Cells lacking RGBARG (*RGBARG−*) form enlarged, flat interior domains unable to generate large macropinosomes and display a geometry-specific defect in engulfing rod-shaped bacteria. IqgC localizes and accumulates strongly on macropinosome and weakly on phagosomes of growth-phase cells. Cells lacking IqgC (*iqgC−*) form larger macropinosomes at a normal frequency and show enhanced phagocytosis efficiency. As with the above three RasGAPs, C2GAP2 also localized on the protrusion sites that further expanded and engulfed to form a macropinosome or phagosome. Interestingly, C2GAP2 remained in the macropinosomes and phagosomes with no active Ras present in these structures, indicating that membrane localization of C2GAP2 does not require the active state of Ras. Moreover, *c2gapB−* cells display no significant differences in the size and intensity of macropinosomes, indicating that C2GAP2 might not play a major role in determining the geometric properties of engulfment. Instead, C2GAP2 might be a general regulator that controls RasB/G activity to modulate macropinocytosis and phagocytosis In addition, our data shows that C2GAP2 is depleted from macropinosomes and phagosomes prior to PIP_3_. PIP_3_ has been demonstrated to function mainly in the formation of both macropinosome and phagosome (27, 31), indicating that C2GAP2 might not play a major role in the later stages of macropinosomes and phagosome, such as acidification or trafficking/recycling of macropinosomes and phagosomes.

*D. discoideum* displays chemotaxis behavior in gradients of both cAMP and folic acid. cAMP-mediated chemotaxis is better understood than folic acid-mediated chemotaxis. Briefly, cAMP engagement of its receptor cAR1 activates heterotrimeric G protein, Gα2/βγ. Free Gβγ2 and Gα2 activate multiple signaling pathways, including PI3K, TorC2, PLA2, and sGC, to mediate chemotaxis (32–35). cAMP-mediated Ras signaling directly or indirectly regulates these four pathways and, more importantly, is the first signaling event in GPCR-mediated signaling pathways that display adaptation (3). Adaptation is a fundamental mechanism by which cells sense an extracellular gradient and establish intracellular polarization of directed cell migration (23). Ras adaptation play a central role in the GPCR-mediated signaling pathways of cAMP-mediated chemotaxis (3). It has been previously shown that both DdNF1- and C2GAP1 mediate Ras adaptation and are required for cAMP-mediated Ras adaptation and chemotaxis in *D. discoideum* (15, 16). The functions of RasGAP proteins rely on their expression in the different life cycle. C2GAP1 is highly expressed only in the early developmental, cAMP-chemotactic stage of social life cycle in *D. discoideum* (15). Different from C2GAP1, C2GAP2 is highly expressed in the vegetative stage. Its expression decreased during the early developmental stage, suggesting its role in vegetative stage when *D. discoideum* cells are chemotactic toward folic acid. Similar to cAMP stimulation, folic acid stimulation triggers a transient, adaptative activation profile of Ras activation in *D. discoideum* (21). Like cells deficient in DdNF1 (*nfa−*) or C2GAP1 (*c2gapA−*), *c2gapB−* cells displayed an increased Ras activity. Folic acid stimulation trigged elevated Ras activation in *c2gapB−* cells. Accordingly, *c2gapB−* cells displayed impaired chemotaxis in a folic acid gradient. More severe defects in chemotaxis were shown when *c2gapB−* cells experienced the gradient at higher concentrations. A concentration-dependent chemotaxis defect is also observed in cAMP-chemotactic *D. discoideum* cells or human neutrophil cells (15, 36), indicating that a concentration-dependent deficiency in chemotaxis might be a general behavior of cells lacking Ras inhibitors.

## Materials and methods

### Cell lines, cell growth and differentiation

Cells expressing the protein of interest were selected by growth in the presence of 20 μg/ml geneticin (Sigma, Steinheim, Germany) or 10 µg/ml blasticidin S, and/or hygromycin (Sigma, Steinheim, Germany) with the requirement of double selection. For differentiation, log-phase vegetative cells were harvested from shaking culture (5×10^6^ cells/ml) and washed twice with developmental buffer (DB: 5 mM Na_2_HPO_4_, 5 mM KH_2_PO_4_, 2 mM MgSO_4_, and 0.2 mM CaCl_2_) before the experiments.

### Establishment of c2gapB- cells.

The *c2gapB* gene of wild-type (WT) cells was disrupted by inserting the blasticidin-resistant (BSR) cassette at nucleotide position 1644 bp~1828 bp including the sequence for the GAP domain. A 5’ fragment of c2gapB was amplified from AX2 genomic DNA by PCR using primers 5’- CGGGGTACCAGTAAAGATGATTTTATGGGATTAG -3’ and 5’- CCCAAGCTTGATACAATCACTTTAGTTGATAATG -3’ and the product was digested with *Kpn*I and *Hind*III. A 3’ fragment was amplified using primers 5’- GGAATTCCATATG CATTATGTCCATTAATTATGTC-3’ and 5’- AAGGAAAAAAGCGGCCGCGAAATATTTTGAAGTATTTTACTC-3’ and was digested with *Nde*I and *Not*I. The PCR products were cloned on opposite sides of the BSR cassette into pLPBLP. The construct was linearized by digestion with *Kpn*I and *Not*I, purified, and transfected into AX2 cells by electroporation. Transformants were selected in D3-T medium (KD Medical) containing 10 µg/ml blasticidin S. Individual colonies were picked from independent transformations.

### Plasmid construction

The coding sequences of C2GAPB, the C2 domain (1-107a.a.), and the GAP domain (108-464a.a.) were cloned into pCV5 plasmid that contains a C-terminal YFP. The C2GAPB coding sequence was also cloned into an pDM353 plasmid that contains a C terminal GFP tag *via* Gateway cloning. Point mutation, to generate the C2GAP2 R199A mutant, were introduced by the method of Quick change.

### Reagents

Anti-pan Ras mouse monoclonal antibody from EMD Millipore (Billerica, MA) was used to detect *D. discoideum* Ras proteins. Anti-GFP monoclonal antibody was from BD Biosciences (San Jose, CA). Anti-GST monoclonal antibodies were from Santa Cruz Biotechnology (Santa Cruz, CA). HRP-conjugated anti-mouse or anti-rabbit IgG was obtained from Jackson ImmunoResearch (West Grove, PA). Alexa 594 was from Invitrogen (Carlsbad, CA). pHrodo was from Thermo Fisher Scientific (Waltham, MA).

### Measurement of GAP activity

For GAP activity measurements,** **the indicated Ras proteins were produced and purified as previously described (37). The MBP-C2GAP2-FL and MBP-GAP domain (AA 108-464) were produced, isolated from *E. coli* Rosetta cells and purified by Maltose Binding Protein Trap (MBPTrap)-affinity column (GE Healthcare). The proteins were eluted in 20 mM Tris, 200 mM NaCl, 5% Glycerol 1 mM β-Mercaptoethanol and 10 mM Maltose, pH7.5 and further purified by size exclusion chromatography (Sephacryl 16/60, GE Healthcare) stored in 50 mM Tris, 50 mM NaCl, 5 mM DTT, and 5 mM MgCl2, pH7,5. The GAP activity was measured as previously reported (13). Briefly, one µM of Ras protein with and without an equal amount of full-length (FL) or GAP domain of C2GAP was incubated with 50 µM of GTP at 20°C in 50 mM Tris pH 7.5, 50 mM NaCl and 5 mM MgCl2. After different lengths of time the GDP content of the samples was analyzed by HPLC (Thermo Ultimate 3000): a reversed phase C18 column was employed to detect GDP and GTP content (in %) as previously described by Eberth and Ahmadian (38). Linear rates of GDP production were plotted (first 4-8 timepoints) using GraFit 5.0 (Erithacus Software).

### Imaging and data processing

Cells were plated and allowed to adhere to the cover glass of a 4-well or a 1-well chamber (Nalge Nunc International, Naperville, IL) for 10 min, and then covered with DB buffer for the live cell imaging experiment. Cells were imaged using a Carl Zeiss LSM780 (Carl Zeiss, Thornwood, NY) with a 60x/NA 1.4 Oil DIC Plan-Apochromatic objective. Images were processed and analyzed by Zen 780 software. Images were further processed in Adobe Photoshop (Adobe Systems, San Jose, CA), and the intensity of the ROI (region of interest) was explored and analyzed with Microsoft Office Excel (Redmond, WA).

### Immunoprecipitation assay

Cells expressing full length (FL) or mutants of C2GAP2 tagged with YFP or GFP were washed twice, resuspended to 8 × 107 in PM buffer (5 μM Na2PO4, 5 μM KH2PO4, and 2 μM MgSO4), and kept on ice before assay. Cells were stimulated with 100 μM folic acid. Aliquots of 0.5 ml cells were lysed at indicated time points with 10 ml immunoprecipitation buffer (IB, 20 mM Tris, pH8.0, 20 mM MgCl2, 10% glycerol, 2 μM Na3VO4, 0.25% NP40, and complete 1× EDTA-free proteinase inhibitor) for 30 min on ice. Cell extracts were centrifuged at 16,000 × g for 10 min at 4°C. Supernatant fractions were collected and incubated with 25 μl anti-GFP agarose beads at 4°C for 2 hours. Beads were washed four times with immunoprecipitation buffer and proteins were eluted by boiling the beads in 50 μl SDS sample buffer.

### Ras activation in macropinocytosis

WT and *c2gapB−* cells were transfected with a plasmid encoding for the active Ras marker Raf1(RBD)-GFP. Single images of vegetative cells were taken using a Zeiss LSM780 confocal microscope. Logarithmically growing wild type and mutant cells from shaken suspension were counted and resuspended in 10 ml fresh HL5 medium at a density of 1×106 cells/ml. After 1 hour of incubation, FITC-dextran (Mw=70,000) was added to the cells at a concentration of 2 mg/ml. Aliquots of 0.5 ml were taken at t=0, 15, 30, 45, 60, 120, and 180 minutes. Cells were spun down and washed once in 1 ml PB and the washed cell pellet was lysed in 40 μl lysis buffer (10 mM Tris pH 8.3, 50 mM KCl, 2.5 mM MgCl2, 0.45% NP40, 0.45% Tween 20). The amount of FITC dextran in the lysate was measured using a fluorometer (470 nm excitation, 520 nm emission). Images were quantified using ImageJ (NIH). Patches of Raf1(RBD)-GFP are essentially discrete and easily identified. The figure shows the mean ± SD of 3 experiments. WT and *c2gapB^-^* cells were transfected with a plasmid encoding for the active Ras marker Raf1(RBD)-GFP. Single images of vegetative cells were taken using a Zeiss LSM800 confocal microscope. Images were quantified using ImageJ (NIH). Patches of Raf1(RBD)-GFP are essentially discrete and easily identified. The fluorescence intensity of each patch was defined as the maximum signal along a line drawn perpendicular to the center of the patch. To correct for differences in expression level, the mean fluorescence intensity of the cytosol of each cell was also determined and the intensity of the patch was divided by the intensity of the cytosol. The size of each Raf1(RBD)-GFP patch was determined using the segmented line tool.

### EZ-TAXIScan chemotaxis assay

*D. discoideum* cells were harvested, washed with DB, and resuspended. Cell migration was recorded at 30 s intervals at 22°C for 60 min in the EZ-TAXIScan chamber. A stable gradient of 100 μM folic acid was established for the assay. Cell migration analysis was performed with DIAS software (24). The extracted data were further analyzed with Excel software.

### Phagocytosis assay and flow cytometry

*K. aerogenes* labeled with pHrodo Red were incubated with *D. discoideum* cells at a 50:1 ratio at 22°C. After incubation, the cells were washed and resuspended in basic buffer (50 mM Tris [pH 8.8] and 150 mM NaCl). The phagocytes and K. aerogenes were distinguished by forward and side scatter (FSC). The appearance of pHrodo in the phagocyte population was monitored as an indicator of K. aerogenes engulfment. The phagocyte cell population characterized by high fluorescence of pHrodo was considered to be the cells that engulfed K. aerogenes. Data acquisition and analysis were done using a FACSort flow cytometer with Cell Quest software (version 3.3) and analyzed using FlowJo (version 10.0.8).

### Measurement of Ras activation in response to folic acid stimulation by pull-down assay

Cells in log-phase growth were harvested from shaking culture (5×10^6^ cells/ml) and washed twice with DB buffer. Next, the cells were resuspended at 2×10^7^ cells/ml with DB buffer and shaken in a shaking flask at 200 rpm for 90 minutes at room temperature. The cells were centrifuged and washed with phosphate buffer (PB: 5 mM Na_2_HPO_4_, 5 mM KH_2_PO_4_). The cells were resuspended with PB at 2×10^8^ cells/ml and sat on ice for 10 min. The cells were transferred to a medical cup and shaken at 200 rpm for 3 min and then stimulated with a final concentration of 10 μM folic acid. Before or after folic acid stimulation at the indicated time points, 0.5 ml aliquots of the cells were lysed in 10 ml immunoprecipitation buffer (IB, 20 mM Tris, pH8.0, 20 mM MgCl_2_, 10% glycerol, 2 mM Na_3_VO_4_, 0.25% NP40, and complete 1X EDTA-free proteinase inhibitor) for 30 min on ice. Cell extracts were centrifuged at 16,000 × *g* for 10 min at 4°C. Aliquots of the supernatants were mixed with same volume of 2X SDS loading buffer for the detection of total Ras protein in the samples. Supernatants were incubated with 25 μl agarose beads conjugated with Arf1-RBD (active Ras biding domain from Arf1) from Cytoskeleton Inc. (Denver, CO) at 4°C for 2 hours. Beads were washed four times with IB. Proteins were eluted by boiling the beads in 25 μl SDS loading buffer. The eluted protein samples and the protein aliquots for total Ras protein were subjected to immunoblotting with Ras antibodies to detect either active or total Ras proteins.

### Fractionation experiment

The fractions of the membrane portion of the cells were obtained by a filter fractionation assay (5). Cells were collected and washed twice with DB buffer. Cells were suspended with PM buffer and mechanically lysed through a filter system with 5 μm pores into PM buffer with or without the indicated concentration of CaCl_2_. The mixtures were centrifuged at 16,000 rpm for1 min. The supernatants were immediately removed. SDS loading buffer was added to the pellets and mixed well. The samples were then subjected to Western blot detection of C2GAP2 with anti-GFP monoclonal antibody.

### Phospholipids binding assay using PIP Strips

Cells were lysed using IB buffer on ice for 30 min and were subjected to centrifugation min at maximum speed for 10 min. The supernatants were moved to a new tube and incubated with PIP Stripd overnight at 4 °C. The PIP Strips were subjected to Western blot detection using anti-GFP monoclonal antibody.

## Data availability statement

The original contributions presented in the study are included in the article/**Supplementary Material**. Further inquiries can be directed to the corresponding author.

## Author contributions

Conceptualization: XX. Investigation: XX, HP, BG, DP, DV, SR, HL. Data analysis: XX, BG, DP, DV. Writing – Original draft: XX. Review & Editing: XX and AK. Funding acquisition: TJ. All authors contributed to the article and approved the submitted version.

## Funding

This work was supported by the NIH Intramural Fund from the National Institute of Allergy and Infectious Diseases, National Institutes of Health.

## Conflict of interest

The authors declare that the research was conducted in the absence of any commercial or financial relationships that could be construed as a potential conflict of interest.

## Publisher’s note

All claims expressed in this article are solely those of the authors and do not necessarily represent those of their affiliated organizations, or those of the publisher, the editors and the reviewers. Any product that may be evaluated in this article, or claim that may be made by its manufacturer, is not guaranteed or endorsed by the publisher.
