# Supplementary figures and images for: C2GAP2 is a common regulator of Ras signaling for chemotaxis, phagocytosis, and macropinocytosis
Source: Front Immunol. 2022 Nov 29;13:1075386. doi: 10.3389/fimmu.2022.1075386 (PMC9745196; doi:10.3389/fimmu.2022.1075386)

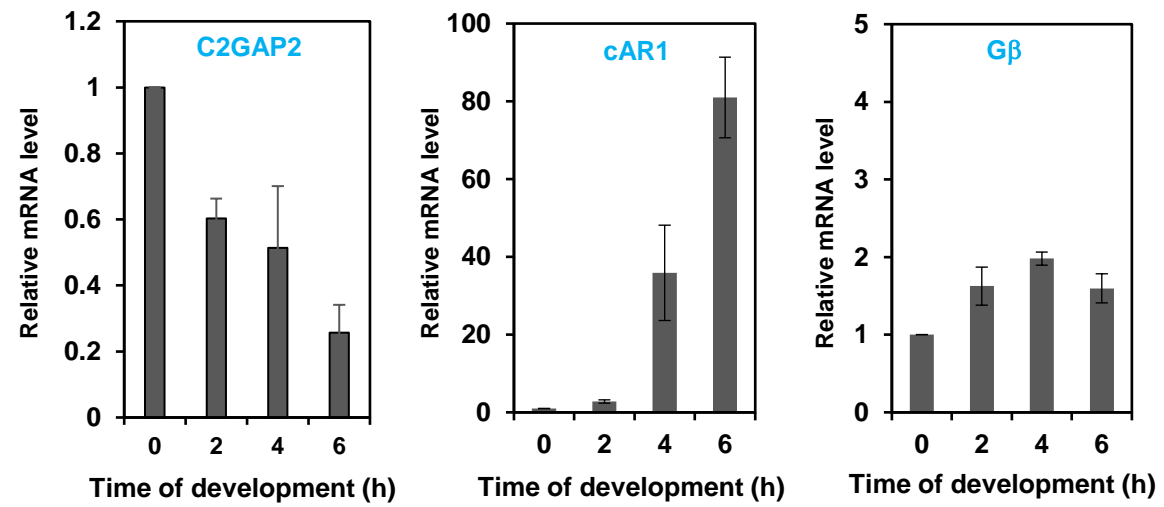

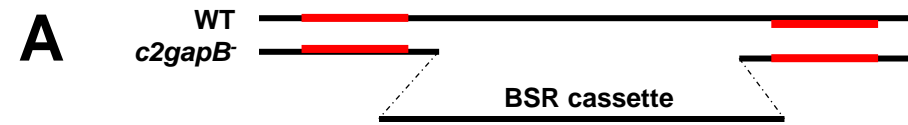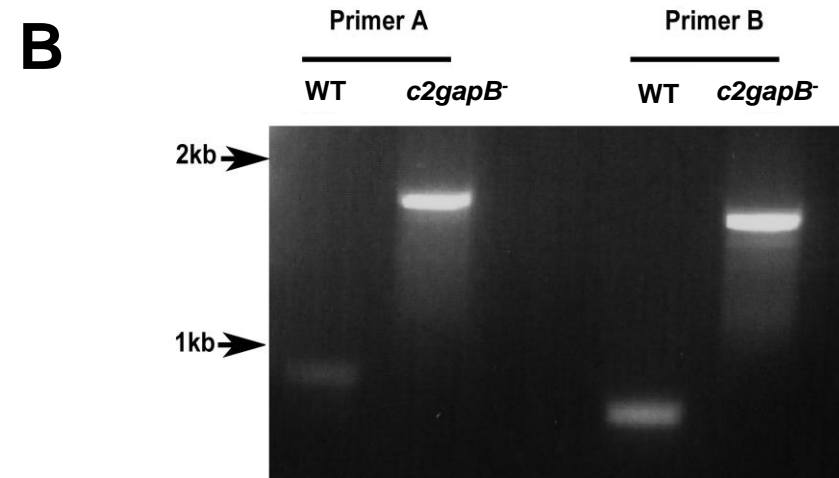

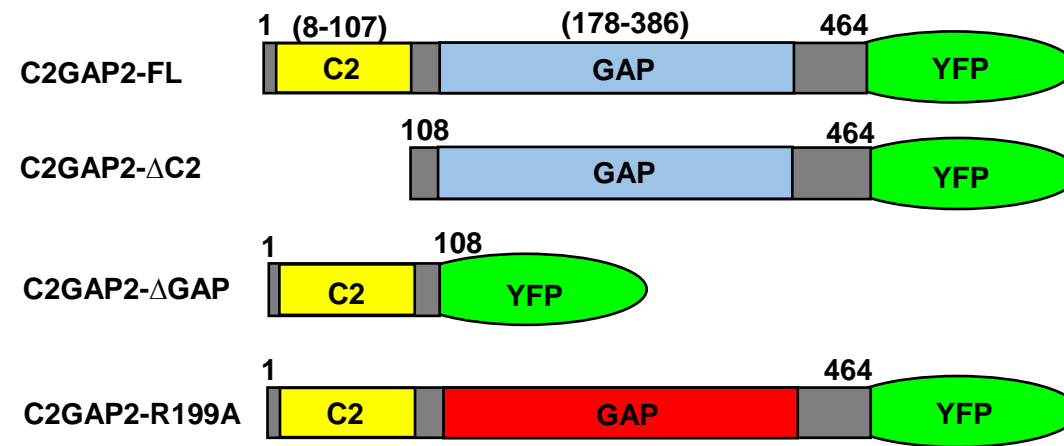

Supplement: Supplementary file 1 [file DataSheet_1.pdf]
