# Supplementary material for: C2GAP2 is a common regulator of Ras signaling for chemotaxis, phagocytosis, and macropinocytosis
Source: Front Immunol. 2022 Nov 29;13:1075386. doi: 10.3389/fimmu.2022.1075386 (PMC9745196; doi:10.3389/fimmu.2022.1075386)
Supplement: Supplementary file 2 [file DataSheet_2.docx]

**SUPPLEMENTAL INFORMATION**

**Fig. S1.** **C2GAP2 is highly expressed in the vegetative stage of *Dictyostelium* cells.** A decreasing expression profile of C2GAP2 during the early developmental stage of *Dictyostelium*. Total mRNA was harvested from wild-type (WT) cells developed in non-nutrient differentiation buffer (DB buffer) with cAMP pulses after 0, 2, 4, and 6 hours. The mRNA levels of the indicated genes were assessed by real-time PCR and normalized, with the mRNA levels at time 0 as 1. The mean ± SD from three independent experiments is shown. The expression of cAR1 and Gβ are also detected as markers of developmental and housekeeping genes, respectively.

**Fig. S2. Establishment of *c2gapB****^−^* **clones.** (A) Scheme shows the strategy of homologous recombination to disrupt the *c2gapB* gene. (B) Sizes of PCR-amplified fragments of WT and *c2gapB*^−^ with two different sets of primers.

**Fig. S3. Domain composition of C2GAP2**. (**A**) Alignment of C2GAP1, human NF1, and p120GAP proteins identifies Arginine residue at 199 of C2GAP2, which is the key residue for the Arginine finger and GAP activity. (**B**) Domain compositions of wild type (WT), the deletion mutants of ΔC2 and ΔGAP, or inactive mutant (R199A) of C2GAP2.

**Supplemental Procedures**

**Real-time PCR detection of gene expression.** Cells were differentiated by exogenous cAMP pulses in DB, and 10^8^ cells were harvested at various times. Total RNA was isolated using TRIzol reagent (Invitrogen) according to the manufacturer's instructions. One μg of DNase-treated RNA was converted to cDNA using the SuperScript first-strand synthesis system (Invitrogen). A 5% volume of the cDNA reaction was used as the template for real-time PCR using a Light-Cycle thermal cycler (Roche Applied Science), and PCR products were detected with SYBR Green I. Real-time PCR conditions were according to the QIAGEN protocol except that the extension temperature was 60°C. cDNA copy number was determined using QuantiTect SYBR Green PCR kit (QIAGEN). The primers used to amplify experimental and control cDNAs were as follows: *c2gapA*: 5′-GATGGAATCATTAACAGTAAAC-3′ and 5′-GAACGTTCAACTTGAAGTAG-3′; cAR1: 5′-TGGGCATCTGTCACATTTATCT-3′ and 5′-GGAACTACATTGCACATCATCAC-3′; Gβ subunit: 5′-CAGTGGTGCTTGTGATGCTA-3′ and 5′-ATGTTGTCGTGGGTGTATTG-3′.

**PCR confirmation of *c2gapB****^−^* **clones.** Genomic DNA of wild-type (WT) and *c2gapB* knock out (*c2gapB^−^*) cells was purified. The c2gapB disruptants were confirmed by PCR analysis using two sets of primers: primer set A: 5’-TTATTTCAATTTTAATGTATGATGAAG-3’ and 5’-AGTAAAGATGATTTTATGGGATTAG-3’; primer set B: 5’-TTATTTCAATTTTAATGTATGATGAAG-3’ and 5’-GGAATTCCATATGCATTATGTCCATTAATTATGTC-3’. The expected sizes of the fragments in WT and c2gapB*^−^* cells were 947 bp and 1622 bp, respectively. Three independent knock-out clones were obtained, and all exhibited the same phenotype.
